# Identification of novel microRNAs regulating HLA-G expression and investigating their clinical relevance in renal cell carcinoma

**DOI:** 10.18632/oncotarget.8567

**Published:** 2016-04-04

**Authors:** Simon Jasinski-Bergner, Adi Reches, Christine Stoehr, Chiara Massa, Evamaria Gonschorek, Stefan Huettelmaier, Juliane Braun, Sven Wach, Bernd Wullich, Verena Spath, Ena Wang, Francesco M. Marincola, Ofer Mandelboim, Arndt Hartmann, Barbara Seliger

**Affiliations:** ^1^ Institute of Medical Immunology, Martin Luther University Halle-Wittenberg, Halle, Germany; ^2^ Faculty of Medicine, The Hebrew University of Jerusalem, Ein Kerem, Jerusalem, Israel; ^3^ Institute of Pathology, Friedrich Alexander University Erlangen-Nuremberg, Erlangen, Germany; ^4^ Institute of Molecular Medicine, Martin Luther University Halle-Wittenberg, Halle, Germany; ^5^ Clinic of Urology, Friedrich Alexander University Erlangen-Nuremberg, Erlangen, Germany; ^6^ Sidra Medical and Research Center, Qatar Foundation, Doha, Qatar

**Keywords:** non-classical HLA class I molecules, microRNA, HLA-G, immune escape, renal cell carcinoma

## Abstract

The non-classical human leukocyte antigen G (HLA-G) is expressed at a high frequency in renal cell carcinoma (RCC) and is associated with a higher tumor grade and a poor clinical outcome. This might be caused by the HLA-G-mediated inhibition of the cytotoxicity of T and NK cells. Therefore a selective targeting of HLA-G might represent a powerful strategy to enhance the immunogenicity of RCC lesions. Recent studies identified a number of HLA-G-regulating microRNAs (miRs) and demonstrated an inverse expression of some of these miRs with HLA-G in RCC *in vitro* and *in vivo*. However, it was postulated that further miRs might exist contributing to the tightly controlled selective HLA-G expression.

By application of a miR enrichment assay (miTRAP) in combination with *in silico* profiling two novel HLA-G-regulatory miRs, miR-548q and miR-628-5p, were identified. Direct interactions of both miRs with the 3′ untranslated region of HLA-G were confirmed with luciferase reporter gene assays. In addition, qPCR analyses and immunohistochemical staining revealed an inverse, expression of miR-628-5p, but not of miR-548q to the HLA-G protein in primary RCC lesions and cell lines. Stable overexpression of miR-548q and miR-628-5p caused a downregulation of HLA-G mRNA and protein. This leads in case of miR-548q to an enhanced NK cell-mediated HLA-G-dependent cytotoxicity, which could be reverted by ILT2 blockade suggesting a control of the immune effector cell activity at least by this miR. The identification of two novel HLA-G-regulatory miRs extends the number of HLA-G-relevant miRs tuning the HLA-G expression and might serve as future therapeutic targets.

## INTRODUCTION

The non-classical HLA class Ib molecule HLA-G exerts some properties, which are distinct from that of classical HLA class Ia molecules, such as limited allelic variability, existence of membrane-bound and soluble isoforms due to alternative splicing and a restricted physiologic expression to mainly immune-privileged tissues and cells [1]. Furthermore, HLA-G can modulate immune cell responses by its interaction with the inhibitory lymphocyte receptors, the immunoglobulin-like transcript (ILT)2, ILT4 and the killer immunoglobulin-like receptor KIR2DL4 present on different immune cell populations [2, 3]. This leads to an inhibition of the immune effector cell-mediated cytotoxicity. In addition, HLA-G induces apoptosis of activated CD8^+^ T cells and suppresses CD4^+^ T cell proliferation in response to allogenic stimulation [4–6].

Under pathophysiologic conditions constitutive HLA-G surface expression was frequently upregulated in hematopoietic and solid tumors including renal cell carcinoma (RCC) [1, 7-9] which could often be correlated to an unfavorable prognosis and poor clinical outcome of tumor patients. In addition, high levels of soluble HLA-G in sera and ascites of tumor patients were correlated with an advanced disease status and higher tumor load [10, 11]. The aberrant expression of HLA-G is regulated by different molecular mechanisms including epigenetic, transcriptional, post-transcriptional as well as post-translational control [12] rather than structural alterations.

Due to the discordant HLA-G mRNA and protein expression often detected in human tumors a post-transcriptional control of HLA-G by miRs has been suggested. Indeed, members of the microRNA (miR)-148 family and miR-133a have been identified to target the 3′ untranslated region (UTR) of HLA-G [8, 13–15] thereby leading to its translational inhibition and/or mRNA degradation. These miRs were able to inhibit HLA-G expression resulting in an increased NK and T cell-mediated killing *in vitro*. Furthermore, the reported HLA-G regulatory miRs exert tumor suppressive activity [16–27]. *In situ* the expression of miR-148a was inversely correlated to HLA-G expression in RCC lesions and cell lines and might have clinical relevance as prognostic biomarker or even as therapeutic target [8, 28, 29]. Based on our previous data other HLA-G regulatory miRs might exist. This is further underlined by Donadi and co-workers suggesting a panel of candidate HLA-G-regulating miRs using *in silico* analyses. However, neither their interaction with the 3′-UTR nor their function has been investigated to prove their post-transcriptional control of HLA-G [30].

In this study two novel HLA-G-regulating miRs, miR-628-5p and miR-548q, were identified using a combination of the miR enrichment technology miTRAP [17, 31] with *in silico* profiling. A direct interaction between these two miRs with the HLA-G 3′-UTR was confirmed by luciferase (luc) reporter gene assays. The consequences of these HLA-G-regulatory miRs were tested in stable miR transfectants followed by determination of HLA-G expression levels and immune recognition by NK cells *in vitro*. Furthermore, the expression of these two novel HLA-G-regulatory miRs was analyzed in both RCC cell lines and primary RCC lesions and correlated to HLA-G expression and clinical parameters.

## RESULTS

### Identification of novel HLA-G-regulatory miRs

**Figure 1:**
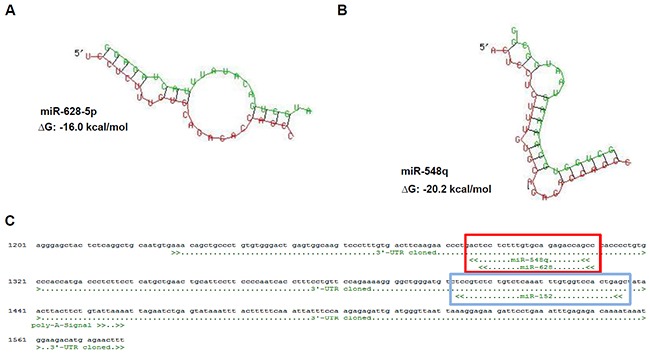
Identification of novel HLA-G regulatory miRs by *in silico* based approach **A, B.** Sequence alignment and prediction of secondary structure including a calculated free energy for the HLA-G 3′-UTR (red) and the novel identified HLA-G regulatory miRs (green) miR-628-5p (A) and miR-548q (B) by usage of the free online data base RNAhybrid [32]. **C.** *In silico* predicted binding sites of the novel identified miRs miR-548q and miR-628-5p (red box), which share the same position at the HLA-G 3′-UTR and is in the 3′ direction localized next to the binding site of the miR-148 family (e.g. miR-152; blue box, [14]).

**Figure 2:**
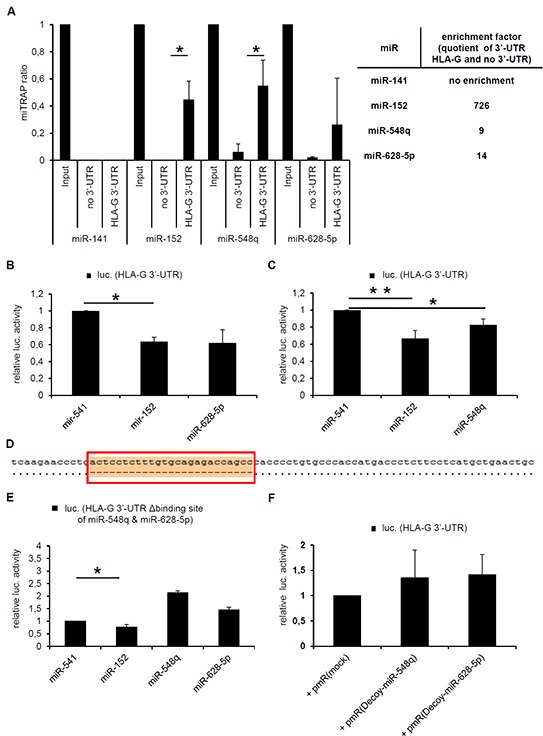
Characterization of the direct interaction between miR-548q, miR-628-5p and the HLA-G 3′-UTR **A.** The results of three miTRAP experiments using *in vitro* transcribed MS2 loop-tagged HLA-G 3′-UTR as bait as described in Materials and Methods followed by qPCR analysis of the miTRAP eluates are shown. The results are expressed as miTRAP ratio [31]. Furthermore, a quotient (enrichment factor) of specifically enriched miRs (HLA-G 3′-UTR as bait) and the non-specifically enriched miRs (mock sequence as bait) was calculated and summarized in the adjacent table. The published HLA-G-regulatory miR-152 served as positive control and showed a higher enrichment with the HLA-G 3′-UTR when compared to the miR-548q and miR-628-5p. The HLA-G non-regulatory miR-141 was expressed in the cell lysate, but not enriched with the HLA-G 3′-UTR. **B, C.** The direct interaction of the miRs miR-548q and miR-628-5p with the HLA-G 3′-UTR was further investigated by luc reporter gene assays as described in Materials and Methods. The results were normalized to the overexpressed HLA-G non-relevant control miR-541, which is not expressed in the applied HEK293T cells and is only reported to play a role in neuronal differentiation [51]. The overexpression of miR-152 served as positive control. In comparison to the negative control miR-541 the overexpression of miR-152, -628-5p and -548q downregulated the luc reporter gene activity of the reporter gene construct encoding the HLA-G 3′-UTR. However, the effect of miR-152 is much stronger than the effect of miR-628-5p or miR-548q. The results were normalized to the overexpressed HLA-G non-relevant control miR-541. **D.** Sequence alignment of the HLA-G wt 3′-UTR (pubmed data base; NM_002127.5) with the HLA-G 3′-UTR (G*010103) including the deletion of the binding site of miR-548q and miR-628 (red box) is visualized after sequencing. The binding site of miR-152 remains unaffected. **E.** No downregulation of the luc reporter gene activity was observed upon a deletion of the binding site for miR-628-5p and miR-548q in the HLA-G 3′-UTR by overexpression of miR-548q and miR-628-5p. Overexpression of miR-152 still down regulates the luc reporter gene activity due to its intact binding site. **F.** A luc reporter gene assay in combination with the wt HLA-G 3′-UTR is shown. Instead of miR expression vectors so called decoy constructs against the miR-548q and miR-628-5p present in the HEK293T cells were transfected, after Haraguchi et al., 2009 [52] leading to a non significant stabilization of the luc reporter gene activity when compared to the respective mock vector.

Although a number of functional HLA-G-regulating miRs has been recently identified [14–16], our previous data suggest the existence of other HLA-G-regulating miRs. The *in silico* screening for HLA-G regulatory miRs by RNA hybrid [32] predicted an interaction between miR-628-5p and miR-548q with the 3′-UTR of HLA-G (Figure 1A, 1B). The predicted binding sites of both novel miRs identified a second hotspot (Figure 1C, red box) for the miR-mediated post-transcriptional control of HLA-G expression next to the binding site of miR-148 family members (Figure 1C, blue box). These novel HLA-G-regulating miRs were confirmed by the miR-specific enrichment from a cell lysate of the HLA-G mRNA^+^/protein^−^ RCC cell line MZ2905RC using the miTRAP technique [31]. Employing a MS2 loop-tagged and *in vitro* transcribed HLA-G 3′-UTR as bait, an enrichment of miR-628-5p and miR-548q was observed by qPCR (Figure 2A). Deep sequencing of the miTRAP eluate also identified both miRs. The abundance of the newly identified miRs for the HLA-G 3′-UTR was lower than for the miR-152, which was taken as positive control for further experiments.

By calculation of a quotient of the specifically enriched miRs with the HLA-G 3′-UTR as bait and the non-specifically enriched miRs with only the MS2 loop sequence as bait results in following affinity of the miRs for the HLA-G 3′-UTR: miR-152 >> miR-548q and miR-628-5p. In contrast, the HLA-G non-relevant miR-141 present in the Input, which served as negative control, was not enriched, demonstrating the specificity of that assay. Furthermore, the direct interaction of miR-628-5p and miR-548q with the HLA-G 3′-UTR was confirmed by luc reporter gene assays. As expected miR-628-5p (Figure 2B) and miR-548q (Figure 2C) expression downregulated the luc reporter gene activity of the HLA-G 3′-UTR containing luc construct normalized to the control miR-541.

Subsequently, the *in silico* predicted target site of miR-628-5p and miR-548q within the HLA-G 3′-UTR was deleted. Figure 2D demonstrates the sequence alignment of the 3′-UTR of HLA-G (NM_002127.5) with the 3′-UTR of HLA-G*010103 including the deletion of the binding site of miR-628-5p and miR-548q after sequencing. The successful deletion of this miR-548q and miR-628-5p binding site caused no downregulation of the luc activity by overexpression of miR-548q and miR-628-5p, while the binding of miR-152 was not affected (Figure 2E). According to Haraguchi and co-authors [52] miR-decoy constructs directed against miR-548q and miR-628-5p were cloned and transfected resulting in a non-significant stabilization of the luc reporter activity when compared to the respective mock control (Figure 2F).

### Expression analysis of miR-548q and miR-628-5p

**Figure 3:**
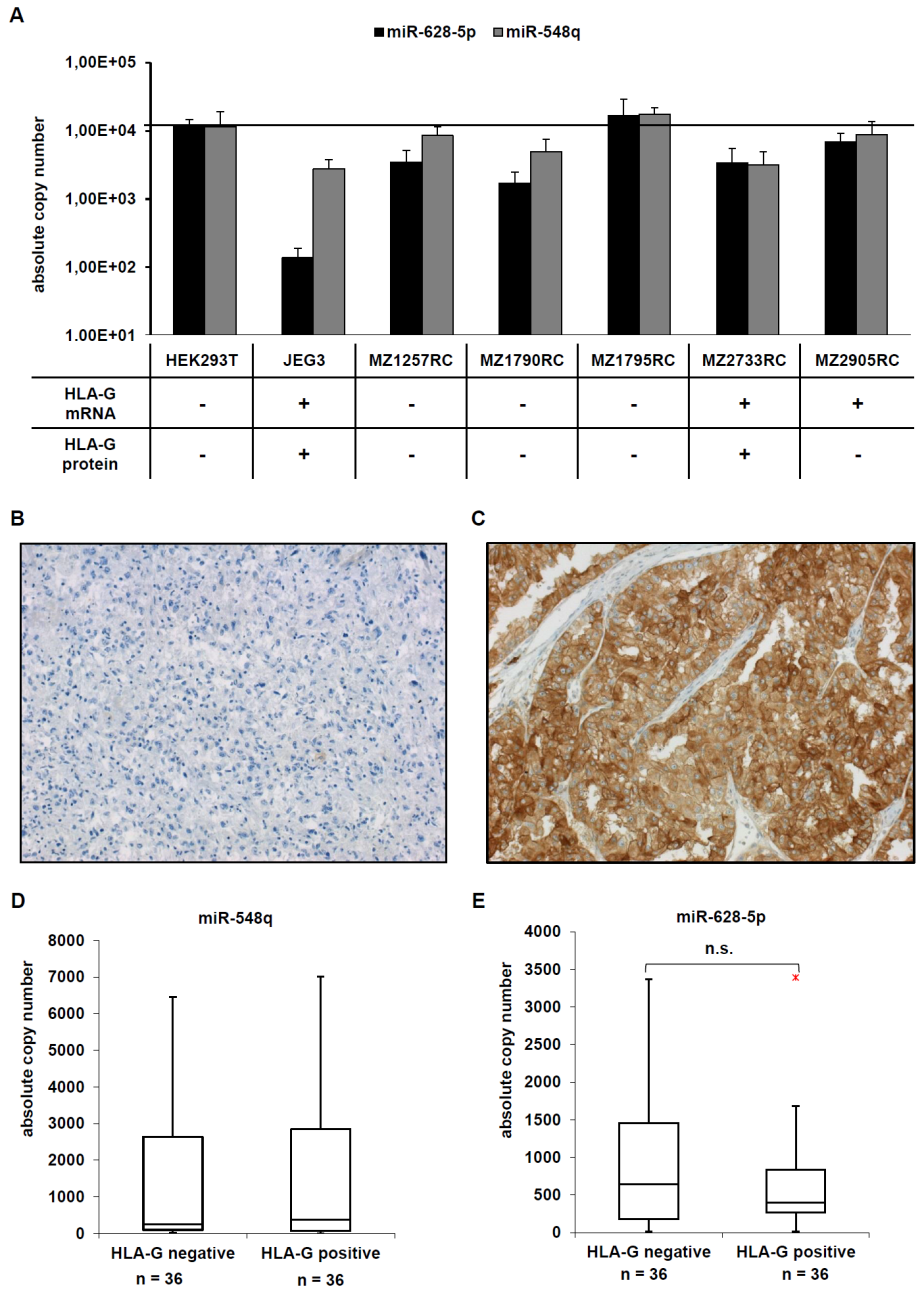
Expression of the novel HLA-G regulating miR-548q and miR-628-5p *in vitro* and *in vivo* **A.** A set of five RCC cell lines, the HEK293T cell line and as HLA-G-positive control the choriocarcinoma cell line JEG-3 were analyzed for the expression of HLA-G, miR-548q and miR-628-5p as described in Material and Methods. The HLA-G expression of the cell lines analysed has already been reported in [8] and summarized in the table below the bar diagram. **B, C.** Two representative immunohistochemical stainings of a HLA-G negative (B) and a HLA-G positive (C) RCC lesion from a tissue microarray consisting of > 450 RCC lesions are shown. **D, E.** The expression of miR-548q (D) and miR-628-5p (E) was determined in 36 HLA-G negative and 36 HLA-G positive RCC lesions by qPCR and results are visualized in Box-Whisker-Plots. The red dot in Figure 3E is an outlayer.

Since both miRs were able to bind to the 3′-UTR of HLA-G their expression was analyzed in five RCC cell lines, from which two were positive for HLA-G mRNA and one for HLA-G protein. The HLA-G-positive choriocarcinoma cell line JEG-3 served as positive control, HEK293T cells as negative control. As shown in Figure 3A, miR-548q and miR-628-5p expression was found at a high frequency in RCC cell lines. Only the miR-628-5p showed an inverse expression pattern to the HLA-G protein with a 100-fold lower expression in HLA-G^+^ JEG-3 cells when compared to other cell lines. It is noteworthy that the HLA-G protein positive cell lines JEG-3 and MZ2733RC also expressed reduced levels of the HLA-G-specific miR-152 (JEG-3) and miR-148A (MZ2733RC) [8], while an inverse expression of miR-628-5p was not detected in MZ2733RC.

### Investigating a clinical relevance of these novel HLA-G regulatory miRs in RCC lesions

To assess whether an inverse correlation between the expression levels of HLA-G and the two novel putative HLA-G-regulatory miRs detected *in vitro* also exists *in situ* 36 selected HLA-G^+^ and 36 HLA-G^−^ RCC lesions were monitored for miR-548q and miR-628-5p expression as recently described for the miRs of the miR-148 family and the miR-133a [8]. A representative immunohistochemical staining for a HLA-G negative (Figure 3B) and a HLA-G positive (Figure 3C) RCC lesion is shown. The expression levels of miR-628-5p, but not of miR-548q were decreased in HLA-G^+^ RCC lesions when compared to HLA-G^−^ RCC lesions (Figure 3D, 3E), but appear not to be of clinical relevance, since no correlation to clinicopathological parameters including the survival of the RCC patients did exist.

### Down regulation of HLA-G expression by HLA-G-specific miRs

**Figure 4:**
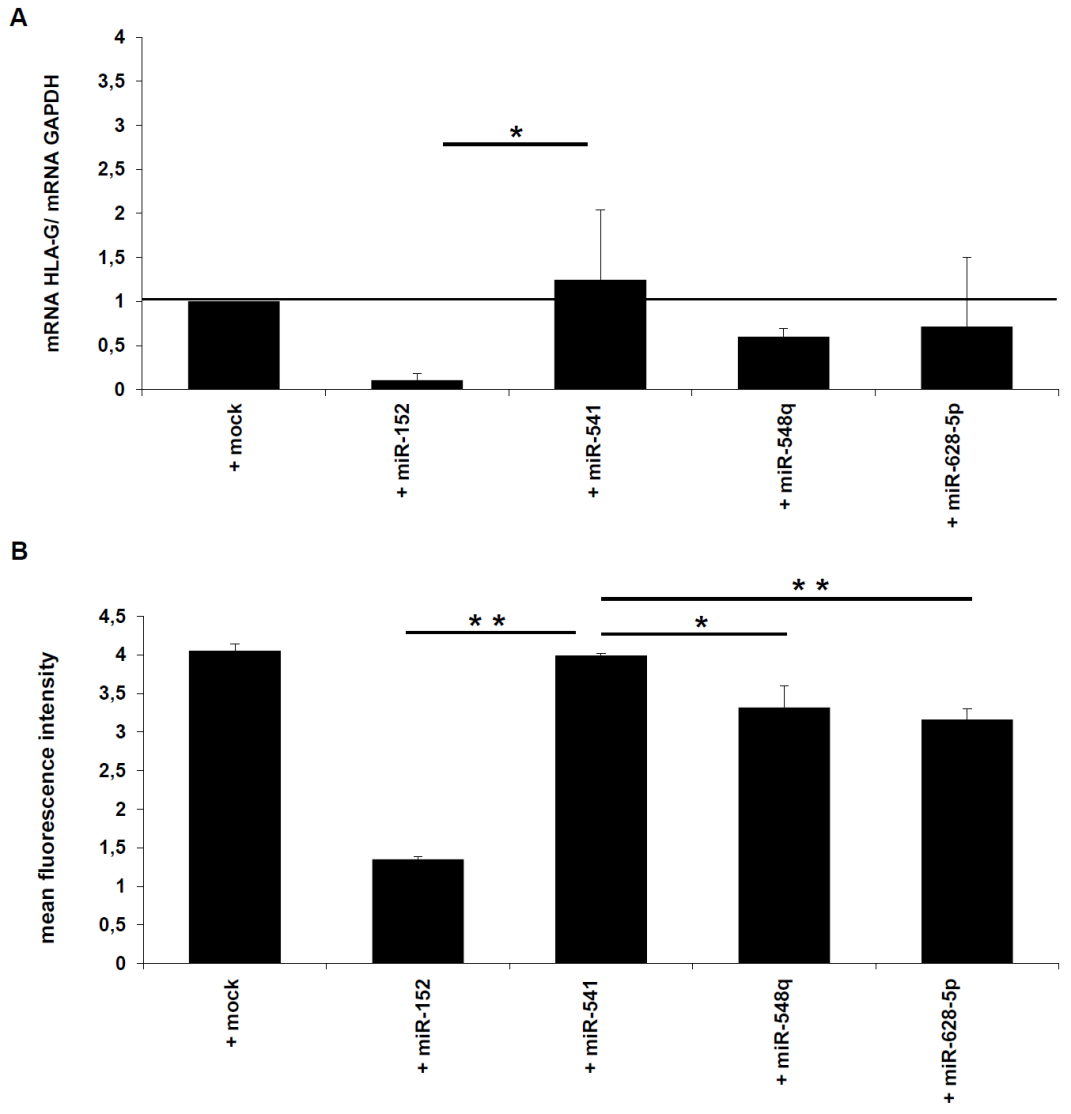
Effects of miR-548q and miR-628-5p on the HLA-G expression *in vitro* **A, B.** HLA-G-positive JEG-3 cells were stably transfected with the expression vectors for miR-152 (as positive control), miR-548q, miR-628-5p, and as negative control miR-541 and the respective mock vector. As described in Materials and Methods the HLA-G mRNA and HLA-G protein expression in the different transfectants was determined by qPCR (A) and flow cytometry (B).

In order to determine whether miR-548q and miR-628-5p affect the HLA-G expression, both miRs were stably transfected into HLA-G^+^ JEG-3 cells, before HLA-G mRNA and protein expression was determined by qPCR and flow cytometry. As shown in Figure 4A and 4B, both miRs inhibit the HLA-G mRNA and protein levels to an equal extent, but in both cases the HLA-G downregulation was weaker than that of miR-152, which served as positive control. As expected, the expression of the HLA-G non-relevant miR-541 as well as the transfection of the mock control had no effect on HLA-G expression.

### Effects of novel HLA-G-specific miRs on the immune response

In order to determine the influence of the miR-548q and miR-628-5p-mediated silencing of HLA-G on the immune effector cell-mediated recognition the HLA class I-negative human B lymphoblastic cell line 721.221 stably transfected with the HLA-G coding sequence with and without the respective HLA-G 3′-UTR was used as model.

**Figure 5:**
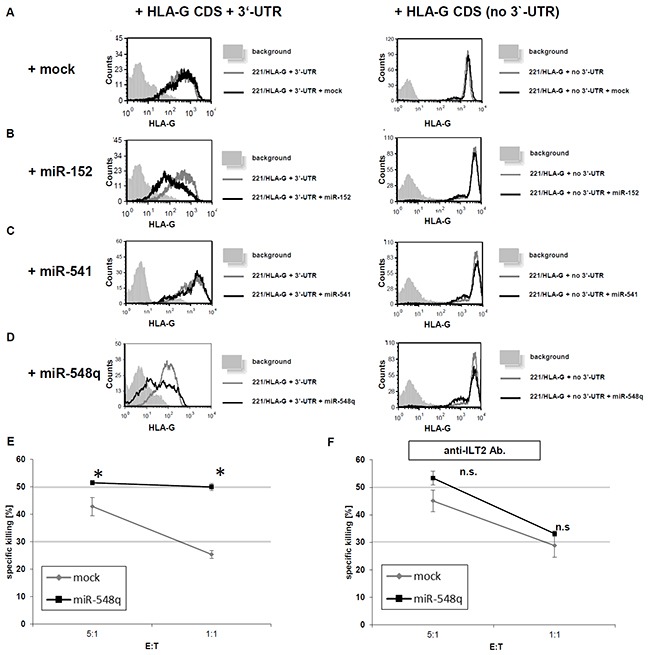
Impact of miR-mediated down regulation of HLA-G expression on the cytotoxicity of NK cells *in vitro* **A–D.** 721.221 cells expressing HLA-G with its 3′-UTR (221/HLA-G + 3′UTR) or expressing HLA-G without the 3′-UTR (221/HLA-G no 3′-UTR) were transduced with the respective mock vector (Figure 5A), the expression vector for miR-152 (positive control; Figure 5B), miR-541 (negative control; Figure 5C) and miR-548q (Figure 5D). The transfectants were analyzed for their HLA-G expression by flow cytometry. The experiments were performed three times. One representative experiment is shown as a histogram. **E.** ^35^S methionine-labelled 721.221/HLA-G + 3′-UTR cells were transduced with an empty vector (mock) or a miR-548q expression vector and then incubated for 5h with ILT2-expressing NK cells. Shown is the relative average killing ± standard derivation of three independent experiments. The HLA-G down regulation upon miR-548q expression caused a statistically significant (*p < 0.05) increase of NK cell mediated cytotoxicity when compared to the mock control, which can even be enhanced at a higher E:T ratio. **F.** From PBMCs freshly isolated NK cells were pretreated with an anti-ILT2 antibody. Due to this blockage of this main HLA-G receptor upon the NK cells no difference in the NK cell mediated cytotoxicity could be observed for the miR-548q overexpressing (HLA-G low) transfectants and the respective mock transfectants (HLA-G high). An increased E:T ratio also increased the cell lysis in both transfectants.

The co-expression of miR-548q and miR-152 in HLA-G^+^ 721.221 cells caused a downregulation of HLA-G surface expression in the transfectants expressing the HLA-G CDS with its 3′-UTR (Figure 5B and 5D), while the transfection of the mock vector or the HLA-G non-relevant miR-541 did not affect HLA-G surface expression (Figure 5A and 5C). Furthermore, HLA-G expression was not downregulated by co-expression of both novel miRs with HLA-G lacking the respective 3′-UTR emphasizing the post-transcriptional gene regulation involving miRs.

The functional role of miR-548q and miR-628-5p in downregulating the HLA-G was determined by analyzing the NK cell-mediated lysis. The miR-548q-mediated downregulation of HLA-G caused an increase in the ILT2^+^ NK cell-mediated cell lysis of the tumor cells, which was statistically significant in the E:T-ratios (1:1 and 5:1) when compared the respective mock transfectants (Figure 5E). These data were further confirmed by an antibody-mediated blocking of the inhibitory NK cell receptor ILT2, which inhibited the HLA-G-dependent lysis (Figure 5F). In Carosella et al., 2011 [53] it is shown that ILT4 is mainly expressed on monocytes, macrophages and dendritic cells, but not on NK cells. Furthermore, the role of KIR2DL4 as inhibitory NK cell receptor is still controversially discussed.

It is noteworthy that overexpression of miR-628-5p did not alter NK cell mediated cytotoxicity despite the downregulation of HLA-G. Thus, miR-548q, but not miR-628-5p is able to revert to the immune escape of HLA-G expressing tumor cells.

## DISCUSSION

Recent studies demonstrated important immunomodulatory effects of HLA-G expression and its role in tumors [1, 8], inflammatory processes and autoimmune diseases [33, 34], transplantation [35, 36] and for recurrent spontaneous abortions of embryos in HLA-G-deficient women [15].

Therefore, a better knowledge of the mechanisms regulating HLA-G expression is of growing interest. Recently, three members of the miR-148 family (miR-148a, -148b, -152) and miR-133a have been shown to control HLA-G expression at the post-transcriptional level [15]. These miRs exert different binding affinities to the HLA-G 3′-UTR [8], which in addition to their abundance contributes to the inhibition of HLA-G expression.

Using different experimental strategies including *in silico* prediction, miTRAP and molecular biological approaches miR-628-5p and miR-548q were identified, which directly interact with the HLA-G 3′-UTR. Both miRs have the same binding site within the HLA-G 3′-UTR identifying a second hotspot of miR binding in the HLA-G 3′-UTR, which is distinct to the one of the miR-148 family. Interestingly, for miR-628-5p a tumor-suppressive function has been described [37] as it has been also shown for other HLA-G regulatory miRs e.g. miR-148a, -148b, -152 and miR-133a [16-18, 21, 25]. These data suggest that miRs could have dual activities by translational repression of the immune inhibitory molecules, such as HLA-G to target and enhance a tumor suppressive phenotype.

Recently, the implementation of miRs or antagomiRs have been suggested as novel therapeutic options against various human diseases including cardiovascular diseases [38, 39], viral infections [40] and tumors [41]. Indeed this strategy is currently tested in animal models or even in clinical trials like Miravirsen (SPC3649/Santaris Pharma A/S) for the treatment of chronic hepatitis C virus infections [42] or MRX34 (Mirna Therapeutics), which recently entered multicenter open-label phase I clinical trials for patients with liver cancer [41].

HLA-G has been shown to exert different immune modulatory effects and to interfere with different immune effector cells *in vitro* and *in vivo* [8, 14, 43]. In this study miR-628-5p and miR-548q expression caused a downregulation of HLA-G expression, and at least miR-548q was able to revert the immune tolerance as already described for miR-152 but to a lower extend. The activity of such miRs as targets could be enhanced by application of respective miR mimics in combination with antibody treatment or in the case of mediating immunologic tolerance after graft transplantation such miRs could be blocked by application of respective antagomiRs.

By the usage of the miTRAP technique a direct interaction of the miR-628-5p and miR-548q with the HLA-G 3′-UTR was demonstrated, which was further confirmed by luc reporter gene assays. By calculation of the enrichment factor the binding affinities of the HLA-G regulatory miRs identified in this study were compared to those of the miR-148 family [8]. Based on this the HLA-G regulatory miRs can be classified into key regulators (miR-152, -148a, -148b) exerting a high affinity for the HLA-G 3′-UTR and fine tuners (miR-133a, -628-5p, -548q) with a much lower affinity for the HLA-G 3′-UTR. The affinity and abundance of the respective HLA-G regulatory miRs in the target tissue should also be considered by targeting these HLA-G regulatory miRs. However, even the fine tuning of HLA-G expression could be accompanied by enhanced NK cell-mediated cytotoxicity as demonstrated for miR-548q.

Although the clinical relevance of HLA-G-specific miRs requires further analysis and should be extended to a larger cohort of RCC lesions a non-significant inverse expression pattern of miR-628-5p in HLA-G^+^ RCC lesions was found. In this context it is noteworthy that a statistically significant inverse expression pattern to the HLA-G protein was only found for miR-148a, but also for the key regulator miR-152 suggesting that other mechanisms are involved in these processes in RCC lesions. This fact should also be considered for potential miR-based anti-HLA-G therapies and for search of potential biomarkers.

## MATERIALS AND METHODS

### Cell lines and tissue culture

The HLA-G negative human embryonal kidney cell line HEK293T and the HLA-G positive choriocarcinoma cell line JEG-3 purchased from the American Type Culture Collection and a set of five established RCC cell lines derived from patients with RCC (MZ1257RC, MZ1790RC, MZ1795RC, MZ2733RC and MZ2905RC) were used. With the exception of JEG-3 cells, which were maintained in RPMI 1640 (Invitrogen), all other cell lines were cultured in Dulbecco's modified Eagles medium (DMEM) (Invitrogen) supplemented with 10 % (V/V) fetal bovine serum (FCS) (PAA, Pasching, Austria), 2 mM L-glutamine (Lonza, Basel, Switzerland) and 1 % penicillin/streptomycin (V/V; PAA).

### Tissue microarray and immunohistochemistry

The construction of the tissue microarray consisting of 453 RCC tumors as well as the immunohistochemical analyses and applied antibodies have been already reported in more detail in Jasinski-Bergner and co-authors, 2015 [8]. Immunohistochemistry (IHC) of 5 μm sections was performed by using the HLA-G-specific mAb [4H84] (Abcam, Cambridge, UK) at a 1:50 dilution generated with the Antibody Diluent (Dako, Hamburg, Germany). As secondary antibody a HRP-linked anti-mouse and the 3,3′-diaminobenzidine (DAB+) substrate chromogen (Dako) was employed.

The evaluation and the pathological diagnostics were performed by the pathologists Prof. A. Hartmann and Dr. V. Spath. The generation of the tissue microarray was performed according the principles expressed in the declaration of Helsinki.

### DNA isolation

Genomic DNA was isolated from different human cell lines using the QIAamp DNA Mini Kit (Qiagen, Hilden, Germany) according the manufacturers' protocol and used as template for PCR amplification and cloning of genes encoding for miR-628-5p and miR-548q. The cloning of expression vectors for miR-152 and miR-541 as well as the HLA-G 3′-UTR containing vectors has been recently reported [8].

### RNA and miR isolation, semi-quantitative and quantitative PCR

Total RNA from cell lines isolated with the TRIzol Reagent (Invitrogen, Carlsbad, CA, USA) according to the manufacturers' instructions followed by DNase I treatment (NEB, Ipswich, MA, USA) and by reverse transcription into cDNA using the RevertAidTM H Minus First Strand cDNA synthesis kit (Fermentas, St. Leon-Rot, Germany). RNA from paraffin-embedded tissue sections was extracted using the MasterPure Complete DNA and RNA Purification Kit (Epicentre Biotechnologies, Madison, WI, USA) according the manufacturers' protocol [8].

For miR-specific cDNA synthesis a miR-specific stem-loop primer was used [44, 45], while for reverse transcription of mRNA oligo dT primers (Fermentas) or the HLA-G-specific reverse primer (5′- TGAGACAGAGACGGAGACAT-3′) were employed. For semi-quantitative RT-PCR the Taq DNA polymerase kit (Invitrogen) and for qPCR the Platinum SYBR Green qPCR SuperMiX-UDG (Invitrogen) were utilized. The reactions were run as triplicates of biologic replicates. The absolute copy numbers of miRs were determined against an external miR-specific TOPO-TA plasmid standard (Invitrogen), which was generated by cloning the respective stem-loop PCR product into this plasmid as recently described [8]. All oligonucleotides used for mRNA and miR expression profiling are listed in the Table 1.

**Table 1:** List of applied oligonucleotides

Primer	Application	Sequence (5′→3′)	Condition	Reference
141-RT-Rct	stem-loop primer	GTCGTATCCAGTGCAGGGTCCGAGGTATTCGCACTGGATACGACCCATCT	42°C	
141 PCR fw	qPCR	GCCCTAACACTGTCTGGTAA	60°C	
152RT-Rct	stem-loop primer	GTCGTATCCAGTGCAGGGTCCGAGGTATTCGCACTGGATACGACCCAAGT	42°C	
152 PCR fw	qPCR	GCCCTCAGTGCATGACAGA	60°C	
541RT-Rct	stem-loop primer	GTCGTATCCAGTGCAGGGTCCGAGGTATTCGCACTGGATACGACAGTCCA	42°C	
541 PCR fw	qPCR	GCCCTGGTGGGCACAGAATC	60°C	
548qRT-Rct	stem-loop primer	GTCGTATCCAGTGCAGGGTCCGAGGTATTCGCACTGGATACGACCCGCCA	42°C	
548q PCR fw	qPCR	GCCCGCTGGTGCAAAAGTAA	60°C	
628-5pRT-Rct	stem-loop primer	GTCGTATCCAGTGCAGGGTCCGAGGTATTCGCACTGGATACGACCCTCTA	42°C	
628-5p PCR fw	qPCR	GCCCATGCTGACATATTTAC	60°C	
stem loop reverse primer	qPCR	GTGCAGGGTCCGAGGT	60°C	
luc (HLA-G) fw	cloning	AAAACTAGTGTGCTGTGGAGAAAGAAGAG	60°C	
luc (HLA-G) rev	cloning	AAAACGCGTAAAGTTCTCATGTCTTCCATTT	60°C	
clonemiR-152fw	cloning	AAACTCGAGTTCTGGGTCCGTTTGGAGT	60°C	
clonemiR-152rev	cloning	AAAGAATTCGTTCTGCCCAGCCCT	60°C	
clonemiR-541fw	cloning	AAACTCGAGAGAATTTCCAGAAGCAACAG	60°C	
clonemiR-541rev	cloning	AAAGAATTCCCAGGATCCCTCAAAGAGTA	60°C	
clonemiR-548qfw	cloning	AAAGAATTCGGCACGTTTCTTTCAACC	60°C	
clonemiR-548qrev	cloning	AAACTCGAGCTGCAAGATGCCGAAATG	60°C	
clonemiR-628-5pfw	cloning	AAACTCGAGGCCATCCCTTACATGCCTTTC	60°C	
clonemiR-628-5prev	cloning	AAAGAATTCACACCTGAGGCGACGGCATCTT	60°C	
miTRAP(HLA-G) fw	cloning	AAAGAATTCAAACAGCTGCCCTGTGT	60°C	
miTRAP(HLA-G) rev	cloning	AAACTCGAGCTCTCAAATTTCAGGAATC	60°C	
HLAGqPCRfw	qRT-PCR/PCR	TTGCTGGCCTGGTTGTCCTT	60°C	[50]
HLAGqPCRrev	qRT-PCR/PCR	TTGCCACTCAGTCCCACACAG	60°C	[50]
Forward GAPDH	qRT-PCR/PCR	CAAGGTCATCCATGACAACTTTG	60°C	Fermentas
Reverse GAPDH	qRT-PCR/PCR	GTCCACCACCCTGTTGCTGTAG	60°C	Fermentas
G.257	PCR	GGAAGAGGAGACACGGAACA	61°C	[46]
G.1225	PCR	TGAGACAGAGACGGAGACAT	61°C	[46]

Abbreviations: Fw: forward; rev: reverse.

The HLA-G mRNA in the RCC cell lines was detected by usage of the primers G.257 and G.1225 first reported in Real et al., 1999 [46] in a semi-qPCR, whereas the HLA-G mRNA in the stable JEG-3 transfectants was determined by another primer pair for qPCR.

### Cloning of the miR expression vectors

The applied miR expression vectors were generated by cloning the respective miR gene from genomic DNA with its flanking regions (+/− approximately 300 bp of flanking regions) into the multiple cloning site of the pmR-m-cherry vector (Clontech, Mountain View, CA, USA). This multiple cloning site is located within the 3′-UTR of a gene encoding for a red fluorescent protein allowing cell sorting of transfected cells afterwards. Additionally, this vector contains a geneticin resistance gene for generation of stable transfectants. The used oligonucleotides are listed in the Table 1.

### Flow cytometry

For flow cytometric analyses the following mAbs were employed: the fluorescein isothiocyanate (FITC)-labeled mouse anti-human HLA-G MEM-G/9 (Exbio) and the respective isotype controls (Beckman Coulter, Krefeld, Germany). The antibodies were used at concentrations recommended by the manufacturer. The measurements were performed with a BD LSRFortessa unit (Becton Dickinson, Heidelberg, Germany).

### Generation of 721.221 cells overexpressing HLA-G

The HLA class I-negative human B lymphoblastic cell line 721.221 [47] was transfected with HLA-G with and without its 3′-UTR. The HLA-G sequence with its 3′-UTR was amplified from JEG-3 cells (accession number NM_002127) using the following primers: Fw HLA-G primer: 5′-CGGGATCCGCCGCCACCATGGTGGTCATGGCGCCC-3′. Rv HLA-G +3′UTR primer: 5′-GGCATTCAAAGTTCTCATGTCTTCCATTTA- 3′. To generate a plasmid expressing HLA-G without its 3′-UTR, the same Fw primer was used, while the following Rv primer was employed: HLA-G 5′-GGAATTCTCAATCTGAGCTCTTCTTTCT-3′. The various fragments were then cloned into the pcDNA3 mammalian expression vector and stably transfected into 721.221 cells by electroporation. The generation of the miR expression vectors or the mock vector has been previously described [48]. Transfectants were selected in complete medium supplemented with 1.8 μg/ml puromycin for selection.

### NK cell isolation and NK cell cytotoxicity assays

NK cells were isolated from healthy donors via MACS separation kit (Miltenyi biotech) and grown in the presence of IL-2 (Peprotech, Hamburg, Germany) and employed in *in vitro* cytotoxicity assays as previously described [49]. For blocking of the ILT2 receptor, NK cells were incubated with 1 μg of anti-ILT2 antibody (clone GHI/75; BioLegend) for 1 h on ice. For each target, the spontaneous ^35^S release of cells not incubated with effector cells was calculated, and maximum ^35^S release was calculated by applying 0.1 M of NaOH to the target cells. The level of ^35^S release was measured after 5 h of incubation with effectors using a β counter TopCount (Packard).

### miR enrichment assay (miTRAP) and miR profiling

To enrich HLA-G-specific miRs the recently published miTRAP method was employed [31]. Briefly, the complete 3′-UTR of HLA-G was cloned upstream of four MS2 loops, *in vitro* transcribed with Riboprobe (Promega, Mannheim, Germany) and used for the enrichment of HLA-G-specific miRs from cell lysates of the RCC cell line MZ2905RC (HLA-G mRNA^+^/ protein^−^). By application of 500 pmol of fusion protein consisting of the MS2 loop and maltose binding protein domains, *in vitro*-transcribed RNAs (HLA-G 3′-UTR and as a mock control a sequence encoding only the four MS2 loops) were loaded on amylose beads (NEB). After washing and blocking steps with yeast tRNA (Promega) and BSA (NEB), the beads were incubated with the cell lysate, then washed with wash buffer before the elution was carried out with maltose solution followed by RNA extraction with TRIzol Reagent (Invitrogen). A specific volume of the cell lysate was used for RNA extraction and applied as an input control. The miR enrichment in the eluates was validated by qPCR.

### Luciferase reporter gene assays

The 3′-UTR of HLA-G was cloned in the pMiR-Report vector (Ambion, Kassel, Germany) with SpeI and MluI restriction enzymes (NEB). On the first day 10^4^ HEK293T cells were seeded into 96 well plates. After 24 h the miR expression plasmids were transfected with Effectene transfection reagent (Qiagen). 48 h after seeding the cells the luciferase reporter gene vectors as well as the β-gal vector for normalization of the transfection efficacy were transfected and 72 h after seeding the cells were lysed in lysis buffer (Promega) and the luc and β-gal activities were determined. As negative control the HLA-G non-relevant miR-541 and as positive control the HLA-G regulatory miR-152 were applied.

### Statistical analyses

Microsoft Excel 2010 (Microsoft Corporation) was used for calculating mean, standard derivation or t-test. For the two sided t-test unequal variances have been selected. The data were significant with a p value < 0.05 and marked with a star (or if lower than 0.005 with two stars). If not otherwise specified the results are expressed as mean of at least three biological replicates including standard deviation.

Mean and median patients' characteristic data and testing statistics on staining associations were performed as described in SPSS (IBM) [8]. Differences were regarded as significant at p<0.05.

## Abbreviations

β-gal: beta-galactosidase
GAPDH: glyceraldehyde-3-phosphate dehydrogenase
HLA-G: human leukocyte antigen G
HRP: horseradish peroxidase
IL: interleukin
ILT: immunoglobulin-like transcript
luc: luciferase
mAb: monoclonal antibody
MFI: mean-specific fluorescence intensity
miR: microRNA
miTRAP: miRNA trapping by RNA in vitro affinity purification
n.d.: not determined
NK: natural killer cell
ntc.: non-template control
sHLA-G: soluble HLA-G
RCC: renal cell carcinoma
UTR: untranslated region
WB: Western blot analysis
WT: wild type

## References

[1] Bukur J, Jasinski S, Seliger B (2012). The role of classical and non-classical HLA class I antigens in human tumors. Semin Cancer Biol.

[2] Shiroishi M, Tsumoto K, Amano K, Shirakihara Y, Colonna M, Braud VM, Allan DS, Makadzange A, Rowland-Jones S, Willcox B, Jones EY, van der Merwe PA, Kumagai I, Maenaka K (2003). Human inhibitory receptors Ig-like transcript 2 (ILT2) and ILT4 compete with CD8 for MHC class I binding and bind preferentially to HLA-G. Proc Natl Acad Sci U S A.

[3] Zhao L, Purandare B, Zhang J, Hantash BM (2013). beta2-Microglobulin-free HLA-G activates natural killer cells by increasing cytotoxicity and proinflammatory cytokine production. Hum Immunol.

[4] Bainbridge DR, Ellis SA, Sargent IL (2000). HLA-G suppresses proliferation of CD4(+) T-lymphocytes. J Reprod Immunol.

[5] Contini P, Ghio M, Poggi A, Filaci G, Indiveri F, Ferrone S, Puppo F (2003). Soluble HLA-A,-B,-C and -G molecules induce apoptosis in T and NK CD8+ cells and inhibit cytotoxic T cell activity through CD8 ligation. Eur J Immunol.

[6] Riteau B, Menier C, Khalil-Daher I, Sedlik C, Dausset J, Rouas-Freiss N, Carosella ED (1999). HLA-G inhibits the allogeneic proliferative response. J Reprod Immunol.

[7] Bukur J, Seliger B (2003). The role of HLA-G for protection of human renal cell-carcinoma cells from immune-mediated lysis: implications for immunotherapies. Semin Cancer Biol.

[8] Jasinski-Bergner S, Stoehr C, Bukur J, Massa C, Braun J, Huttelmaier S, Spath V, Wartenberg R, Legal W, Taubert H, Wach S, Wullich B, Hartmann A, Seliger B (2015). Clinical relevance of miR-mediated HLA-G regulation and the associated immune cell infiltration in renal cell carcinoma. Oncoimmunology.

[9] Bukur J, Rebmann V, Grosse-Wilde H, Luboldt H, Ruebben H, Drexler I, Sutter G, Huber C, Seliger B (2003). Functional role of human leukocyte antigen-G up-regulation in renal cell carcinoma. Cancer Res.

[10] Nuckel H, Rebmann V, Durig J, Duhrsen U, Grosse-Wilde H (2005). HLA-G expression is associated with an unfavorable outcome and immunodeficiency in chronic lymphocytic leukemia. Blood.

[11] Singer G, Rebmann V, Chen YC, Liu HT, Ali SZ, Reinsberg J, McMaster MT, Pfeiffer K, Chan DW, Wardelmann E, Grosse-Wilde H, Cheng CC, Kurman RJ, Shih Ie M (2003). HLA-G is a potential tumor marker in malignant ascites. Clin Cancer Res.

[12] Rouas-Freiss N, Moreau P, LeMaoult J, Carosella ED (2014). The dual role of HLA-G in cancer. J Immunol Res.

[13] Amiot L, Ferrone S, Grosse-Wilde H, Seliger B (2011). Biology of HLA-G in cancer: a candidate molecule for therapeutic intervention?. Cell Mol Life Sci.

[14] Manaster I, Goldman-Wohl D, Greenfield C, Nachmani D, Tsukerman P, Hamani Y, Yagel S, Mandelboim O (2012). MiRNA-mediated control of HLA-G expression and function. PLoS One.

[15] Wang X, Li B, Wang J, Lei J, Liu C, Ma Y, Zhao H (2012). Evidence that miR-133a causes recurrent spontaneous abortion by reducing HLA-G expression. Reprod Biomed Online.

[16] Jasinski-Bergner S, Mandelboim O, Seliger B (2014). The role of microRNAs in the control of innate immune response in cancer. J Natl Cancer Inst.

[17] Jasinski-Bergner S, Stehle F, Gonschorek E, Kalich J, Schulz K, Huettelmaier S, Braun J, Seliger B (2014). Identification of 14-3-3beta gene as a novel miR-152 target using a proteome-based approach. J Biol Chem.

[18] Kawakami K, Enokida H, Chiyomaru T, Tatarano S, Yoshino H, Kagara I, Gotanda T, Tachiwada T, Nishiyama K, Nohata N, Seki N, Nakagawa M (2012). The functional significance of miR-1 and miR-133a in renal cell carcinoma. Eur J Cancer.

[19] Kinoshita T, Nohata N, Fuse M, Hanazawa T, Kikkawa N, Fujimura L, Watanabe-Takano H, Yamada Y, Yoshino H, Enokida H, Nakagawa M, Okamoto Y, Seki N (2012). Tumor suppressive microRNA-133a regulates novel targets: moesin contributes to cancer cell proliferation and invasion in head and neck squamous cell carcinoma. Biochem Biophys Res Commun.

[20] Kojima S, Chiyomaru T, Kawakami K, Yoshino H, Enokida H, Nohata N, Fuse M, Ichikawa T, Naya Y, Nakagawa M, Seki N (2012). Tumour suppressors miR-1 and miR-133a target the oncogenic function of purine nucleoside phosphorylase (PNP) in prostate cancer. Br J Cancer.

[21] Moriya Y, Nohata N, Kinoshita T, Mutallip M, Okamoto T, Yoshida S, Suzuki M, Yoshino I, Seki N (2012). Tumor suppressive microRNA-133a regulates novel molecular networks in lung squamous cell carcinoma. J Hum Genet.

[22] Song YX, Yue ZY, Wang ZN, Xu YY, Luo Y, Xu HM, Zhang X, Jiang L, Xing CZ, Zhang Y (2011). MicroRNA-148b is frequently down-regulated in gastric cancer and acts as a tumor suppressor by inhibiting cell proliferation. Mol Cancer.

[23] Tsuruta T, Kozaki K, Uesugi A, Furuta M, Hirasawa A, Imoto I, Susumu N, Aoki D, Inazawa J (2011). miR-152 is a tumor suppressor microRNA that is silenced by DNA hypermethylation in endometrial cancer. Cancer Res.

[24] Xiang Y, Ma N, Wang D, Zhang Y, Zhou J, Wu G, Zhao R, Huang H, Wang X, Qiao Y, Li F, Han D, Wang L, Zhang G, Gao X (2014). MiR-152 and miR-185 co-contribute to ovarian cancer cells cisplatin sensitivity by targeting DNMT1 directly: a novel epigenetic therapy independent of decitabine. Oncogene.

[25] Zhou X, Zhao F, Wang ZN, Song YX, Chang H, Chiang Y, Xu HM (2012). Altered expression of miR-152 and miR-148a in ovarian cancer is related to cell proliferation. Oncol Rep.

[26] Zhou Y, Wu D, Tao J, Qu P, Zhou Z, Hou J (2013). MicroRNA-133 inhibits cell proliferation, migration and invasion by targeting epidermal growth factor receptor and its downstream effector proteins in bladder cancer. Scand J Urol.

[27] Zhu C, Li J, Ding Q, Cheng G, Zhou H, Tao L, Cai H, Li P, Cao Q, Ju X, Meng X, Qin C, Hua L, Shao P, Yin C (2013). miR-152 controls migration and invasive potential by targeting TGFalpha in prostate cancer cell lines. Prostate.

[28] Luck ME, Muljo SA, Collins CB (2015). Prospects for Therapeutic Targeting of MicroRNAs in Human Immunological Diseases. J Immunol.

[29] Zaleska K (2015). miRNA - Therapeutic tool in breast cancer? Where are we now?. Rep Pract Oncol Radiother.

[30] Porto IO, Mendes-Junior CT, Felicio LP, Georg RC, Moreau P, Donadi EA, Chies JA, Castelli EC (2015). MicroRNAs targeting the immunomodulatory HLA-G gene: a new survey searching for microRNAs with potential to regulate HLA-G. Mol Immunol.

[31] Braun J, Misiak D, Busch B, Krohn K, Huttelmaier S (2014). Rapid identification of regulatory microRNAs by miTRAP (miRNA trapping by RNA in vitro affinity purification). Nucleic Acids Res.

[32] Rehmsmeier M, Steffen P, Hochsmann M, Giegerich R (2004). Fast and effective prediction of microRNA/target duplexes. RNA.

[33] Rothe K, Quandt D, Schubert K, Rossol M, Klingner M, Jasinski-Bergner S, Scholz R, Seliger B, Pierer M, Baerwald C, Wagner U (2016). Latent CMV infection in rheumatoid arthritis increases frequencies of cytolytic LIR-1+ CD8+ T cells. Arthritis Rheumatol.

[34] Veit TD, Chies JA (2009). Tolerance versus immune response -- microRNAs as important elements in the regulation of the HLA-G gene expression. Transpl Immunol.

[35] Brugiere O, Thabut G, Krawice-Radanne I, Rizzo R, Dauriat G, Danel C, Suberbielle C, Mal H, Stern M, Schilte C, Pretolani M, Carosella ED, Rouas-Freiss N (2015). Role of HLA-G as a predictive marker of low risk of chronic rejection in lung transplant recipients: a clinical prospective study. Am J Transplant.

[36] LeMaoult J, Daouya M, Wu J, Loustau M, Horuzsko A, Carosella ED (2013). Synthetic HLA-G proteins for therapeutic use in transplantation. FASEB J.

[37] Schulte JH, Marschall T, Martin M, Rosenstiel P, Mestdagh P, Schlierf S, Thor T, Vandesompele J, Eggert A, Schreiber S, Rahmann S, Schramm A (2010). Deep sequencing reveals differential expression of microRNAs in favorable versus unfavorable neuroblastoma. Nucleic acids research.

[38] Thum T, Gross C, Fiedler J, Fischer T, Kissler S, Bussen M, Galuppo P, Just S, Rottbauer W, Frantz S, Castoldi M, Soutschek J, Koteliansky V, Rosenwald A, Basson MA, Licht JD (2008). MicroRNA-21 contributes to myocardial disease by stimulating MAP kinase signalling in fibroblasts. Nature.

[39] Xu LJ, Ouyang YB, Xiong X, Stary CM, Giffard RG (2015). Post-stroke treatment with miR-181 antagomir reduces injury and improves long-term behavioral recovery in mice after focal cerebral ischemia. Exp Neurol.

[40] Israelow B, Mullokandov G, Agudo J, Sourisseau M, Bashir A, Maldonado AY, Dar AC, Brown BD, Evans MJ (2014). Hepatitis C virus genetics affects miR-122 requirements and response to miR-122 inhibitors. Nat Commun.

[41] Hydbring P, Badalian-Very G (2013). Clinical applications of microRNAs. F1000Res.

[42] Oprea II, Viola JR, Moreno PM, Simonson OE, Rodin S, Teller N, Tryggvason K, Lundin KE, Girnita L, Smith CI (2014). Repeatable, Inducible Micro-RNA-Based Technology Tightly Controls Liver Transgene Expression. Mol Ther Nucleic Acids.

[43] Loumagne L, Baudhuin J, Favier B, Montespan F, Carosella ED, Rouas-Freiss N (2014). In vivo evidence that secretion of HLA-G by immunogenic tumor cells allows their evasion from immunosurveillance. Int J Cancer.

[44] Chen C, Ridzon DA, Broomer AJ, Zhou Z, Lee DH, Nguyen JT, Barbisin M, Xu NL, Mahuvakar VR, Andersen MR, Lao KQ, Livak KJ, Guegler KJ (2005). Real-time quantification of microRNAs by stem-loop RT-PCR. Nucleic acids research.

[45] Varkonyi-Gasic E, Wu R, Wood M, Walton EF, Hellens RP (2007). Protocol: a highly sensitive RT-PCR method for detection and quantification of microRNAs. Plant methods.

[46] Real LM, Cabrera T, Collado A, Jimenez P, Garcia A, Ruiz-Cabello F, Garrido F (1999). Expression of HLA G in human tumors is not a frequent event. Int J Cancer.

[47] Shimizu Y, DeMars R (1989). Demonstration by class I gene transfer that reduced susceptibility of human cells to natural killer cell-mediated lysis is inversely correlated with HLA class I antigen expression. Eur J Immunol.

[48] Tsukerman P, Stern-Ginossar N, Gur C, Glasner A, Nachmani D, Bauman Y, Yamin R, Vitenshtein A, Stanietsky N, Bar-Mag T, Lankry D, Mandelboim O (2012). MiR-10b downregulates the stress-induced cell surface molecule MICB, a critical ligand for cancer cell recognition by natural killer cells. Cancer Res.

[49] Mandelboim O, Reyburn HT, Vales-Gomez M, Pazmany L, Colonna M, Borsellino G, Strominger JL (1996). Protection from lysis by natural killer cells of group 1 and 2 specificity is mediated by residue 80 in human histocompatibility leukocyte antigen C alleles and also occurs with empty major histocompatibility complex molecules. J Exp Med.

[50] Pavan L, Tarrade A, Hermouet A, Delouis C, Titeux M, Vidaud M, Therond P, Evain-Brion D, Fournier T (2003). Human invasive trophoblasts transformed with simian virus 40 provide a new tool to study the role of PPARgamma in cell invasion process. Carcinogenesis.

[51] Zhang J, Zhang J, Liu LH, Zhou Y, Li YP, Shao ZH, Wu YJ, Li MJ, Fan YY, Shi HJ (2011). Effects of miR-541 on neurite outgrowth during neuronal differentiation. Cell biochemistry and function.

[52] Haraguchi T, Ozaki Y, Iba H (2009). Vectors expressing efficient RNA decoys achieve the long-term suppression of specific microRNA activity in mammalian cells. Nucleic acids research.

[53] Carosella ED, Gregori S, LeMaoult J (2011). The tolerogenic interplay(s) among HLA-G, myeloid APCs, and regulatory cells. Blood.

